# Bid Expression Network Controls Neuronal Cell Fate During Avian Ciliary Ganglion Development

**DOI:** 10.3389/fphys.2018.00797

**Published:** 2018-06-29

**Authors:** Sophie Koszinowski, Veronica La Padula, Frank Edlich, Kerstin Krieglstein, Hauke Busch, Melanie Boerries

**Affiliations:** ^1^Department of Molecular Embryology, Institute of Anatomy and Cell Biology, Albert-Ludwigs-University Freiburg, Freiburg, Germany; ^2^Faculty of Biology, Albert-Ludwigs-University Freiburg, Freiburg, Germany; ^3^Institute for Biochemistry and Molecular Biology, and Centre for Biological Signalling Studies BIOSS, Albert-Ludwigs-University Freiburg, Freiburg, Germany; ^4^Institute of Molecular Medicine and Cell Research, Albert-Ludwigs-University Freiburg, Freiburg, Germany; ^5^Luebeck Institute for Experimental Dermatology, University of Lübeck, Lübeck, Germany; ^6^Institute for Cardiogenetics, University of Lübeck, Lübeck, Germany; ^7^German Cancer Consortium (DKTK), Freiburg, Germany; ^8^German Cancer Research Center (DKFZ), Heidelberg, Germany

**Keywords:** network inference, protein interaction network, ontogenetic cell death, neuronal development, BID, time series analysis

## Abstract

Avian ciliary ganglion (CG) development involves a transient execution phase of apoptosis controlling the final number of neurons, but the time-dependent molecular mechanisms for neuronal cell fate are largely unknown. To elucidate the molecular networks regulating important aspects of parasympathetic neuronal development, a genome-wide expression analysis was performed during multiple stages of avian CG development between embryonic days E6 and E14. The transcriptome data showed a well-defined sequence of events, starting from neuronal migration via neuronal fate cell determination, synaptic transmission, and regulation of synaptic plasticity to growth factor associated signaling. In particular, we extracted a neuronal apoptosis network that characterized the cell death execution phase at E8/E9 and apoptotic cell clearance at E14 by combining the gene time series analysis with network synthesis from the chicken interactome. Network analysis identified TP53 as key regulator and predicted involvement of the BH3 interacting domain death agonist (BID). A virus-based RNAi knockdown approach *in vivo* showed a crucial impact of BID expression on the execution of ontogenetic programmed cell death (PCD). In contrast, Bcl-XL expression did not impact PCD. Therefore, BID-mediated apoptosis represents a novel cue essential for timing within CG maturation.

## Introduction

The chicken ciliary ganglion (CG) houses a homogenous neuronal population undergoing synchronized changes during embryonic development (Landmesser and Pilar, 1974). Due to its accessibility and ease of experimental manipulation both *in vitro* and *in vivo* (Dryer, 1994), the CG has since served as a classical model to study parasympathetic development and synapse formation. Recently, even optogenetic manipulation of the CG has been established in a study investigating presynaptic terminals (Stanchev and Sargent, 2011; Egawa et al., 2013; Ruffinatti et al., 2013; Simpson et al., 2013; Zamburlin et al., 2013).Precursors of CG neurons migrate from the cranial neural crest to form the ganglion by embryonic day (E) 4, after which they become postmitotic and set out to express neuronal marker proteins (Rohrer and Thoenen, 1987). Between E6 and E10, efferent and afferent synaptic connections are established, accompanied by a massive wave of neuronal programmed cell death (PCD) (Landmesser and Pilar, 1974; Narayanan and Narayanan, 1981) and these processes are finished by E14 (Landmesser and Pilar, 1978). Nevertheless, the understanding of the molecular mechanisms controlling important developmental events in the CG still remain limited and comprehensive information about regulatory networks guiding and shaping these events over time are needed to better understand the basic principles of neural development and differentiation.

Here, we report *in vivo* gene expression analysis of CG development between E6 and E14, focusing on the transient regulation of apoptosis as the key event for neuronal cell fate decisions. Time series analysis identified a sequence of differentiation stages with TGF-beta, Notch and neurogenesis related genes becoming particularly activated after a transient phase of apoptosis regulation around E8 and E9. We adapted a novel subgraph extraction method for the chicken protein interactome comprising 9156 nodes and 129,972 edges to construct an apoptosis network that captured the molecular basis of transient neuron cell death in the CG. By means of network centrality we predicted the importance of central genes, like TP53 and TP63 (tumor proteins p53 and p63), as well as of peripheral input/output effectors, such as RARB (Retinoic Acid Receptor, Beta) and BID (BH3 Interacting Domain Death Agonist), a direct target of TP53. We verified experimentally the impact of the latter using an RNAi-mediated knockdown approach. Inhibition of either BID or BCL-XL (apoptosis regulator Bcl-XL /Bcl-2-Like Protein 1 with gene symbol BCL2L1), members of the Bcl-2 protein family with respective pro- and anti-apoptotic function (Boise et al., 1993; Wang et al., 1996), increased the number of CG neurons, or, in the case of BCL-XL knockdown, had no impact on neuronal apoptosis. Taken together, dynamic transcriptome analysis in combination with protein network reconstruction and experimental validation confirmed sequential pathway activity and transient activation of apoptosis to be key during neuronal CG development and confirmed an essential role of BID expression as a novel player in this process.

## Materials and Methods

### Microarrays

Ciliary ganglia were dissected from E6-E10 and E14 chicken embryos. For each analyzed time point, ganglia from at least 40 embryos were pooled for RNA extraction using the Qiagen RNeasy micro kit (Qiagen, Hilden, Germany) according to manufacturer’s instructions. Four replicate sample of each time point were obtained. For transcriptome analysis, 4x44K Agilent (Santa Clara, CA, United States) whole transcriptome arrays were used. The raw intensity reads for each array were background corrected by spatial detrending and subsequently lowess normalized for comparison across arrays using the R limma package (Smyth, 2005). Batch correction was done using an empirical Bayesian approach (Chen et al., 2011) accounting for experiment and time batches. Subsequent analysis considered only genes having a corresponding gene symbol annotation. If multiple probes mapped to the same gene symbol, the probe with the largest IQR was chosen and all others discarded, leaving 13441 transcripts for analysis. Microarray data has been deposited in GEO under the accession ID GSE65426.

### Principal Component Analysis

Principal component analysis (PCA) was performed on the normalized, batch corrected and gene symbol filtered expression data using the prcomp function from the R environment. Data points have been grouped according to time and enveloped by a convex hull to guide the eye.

### Gene Set Enrichment Analysis

Functional annotation of differential gene regulation along the developmental time axis was performed using Gene set enrichment analysis (GSEA) (Luo et al., 2009). For gene set definitions we used the Gene Ontology (GO) for chicken from GO Consortium (built July 2014), which maps 11,572 genes onto 11,062 different ontology terms. We considered only gene sets having three or more members. To assess the significance in differential regulation we used a two-sample *t*-test and Stouffer’s method to summarize individual *p*-values. Raw GSEA *p*-values were corrected for multiple testing using the Benjamini-Hochberg method that controls the false discovery rate (FDR).

### Network Inference

The apoptosis interaction network was calculated from the G. gallus String network (Version 9.1, confidence score > 0.4) (Franceschini et al., 2013). We manually added the TP53 interactome to the network, considering only interactions that (i) were found both in human and mouse and (ii) matched a chicken homolog gene, The dNetFind function from the R dnet package (Fang and Gough, 2014) was used to calculate the apoptosis subnetwork. For this we selected preferentially for genes with a transient fold change regulation at E8 and E9 and that were related to apoptosis. In detail, we fitted a second order polynomial to each gene timeseries and used the coefficient of the quadratic term as score. Next, we weighted all scores by adding +2 or -2 to each score, depending on whether or not a gene belonged to the GO categories GO:1900119 (positive regulation of execution phase of apoptosis), GO:0042771 (intrinsic apoptotic signaling pathway in response to DNA damage by p53 class mediator), GO:0097191 (extrinsic apoptotic signaling pathway), or GO:0043065 (positive regulation of apoptotic process). Network modularization was calculated using a spin-model with simulated annealing from the R igraph package (Csardi and Nepusz, 2006). A conditional hypergeometric test was performed to calculate the GO enrichment within the network modules using all gene nodes of the chicken interactome as background.

### Quantitative Real-Time PCR

RNA was isolated from ciliary ganglia using the RNeasy micro kit (Qiagen, Hilden, Germany), according to the manufacturer’s instructions. RNA was reverse transcribed to cDNA with the RevertAid First Strand cDNA Synthesis Kit (Thermo Fisher Scientific, Schwerte, Germany). Quantitative RT-PCR analysis was performed with the MyiQ^TM^ (BIO-RAD, München, Germany) and the GoTaq^®^ qPCR Master Mix (Promega, Mannheim, Germany) with 5 ng of cDNA template in a 12.5 μl reaction mixture. BID and BCL-XL expression levels were assessed at three different time points during development (E7, E9, and E14), with three independent biological replicates for every time point and three technical replicates. The expression levels were normalized to chicken GAPDH and results were analyzed with the comparative CT method. Data are expressed as 2^-ΔΔC_T_^ normalized to GAPDH and presented as fold change of expression values at E7 and E9 relative to the expression value at E14.

### Production of shRNA Virus

RCAS(BP)B virus carrying an shRNA constructs against BID and BCL-XL were produced according to (Das et al., 2006). The primers used for the BID hairpin in the cloning oligos were: shBID forward: gagaggtgctgctgagcgggatgctctctgttacgatagtagtgaagccacagatgta and shBID reverse: attcaccaccactaggcaagatgctctctgttacgatagttacatctgtggcttcact. The primers for shBCL-XL were: shBCL-XL forward: gagaggtgctgctgagcgggcaggtagtgaatgaactctttagtgaagccacagatgta and shBCL-XL reverse: attcaccaccactaggcaagcaggtagtgaatgaactctttacatctgtggcttcact. DF-1 cells were transfected with the RCAS(BP)B shBID and RCAS(BP)BshBCL-XL plasmids as well as RCAS(BP)B plasmids without an shRNA insert using Lipofectamine 2000 (Invitrogen, Life Technologies, Darmstadt, Germany). Virus was concentrated from the culture medium using Amicon ultra-4 centrifugal filter units (Merck Millipore, Darmstadt, Germany) with a 100 kDa cutoff. Virus stock aliquots were stored at -20° until use.

### Injection of Virus Into Chick Embryos

Fertilized white leghorn chicken eggs (*Gallus gallus domesticus*) were obtained from a local farm and incubated at 38.5°C and 70% humidity. Embryos were staged according to (Hamburger and Hamilton, 1992).Virus stock was mixed with a vital dye (Fast Green, Sigma) and backfilled into a glass capillary. The virus was injected into the neural tube of HH9 chicken embryos at the level of the mesencephalon, where the progenitors of the CG neurons will delaminate (Narayanan and Narayanan, 1981). Embryos were either injected with RCAS(BP)BshBID, RCAS(BP)BshBCL-XL or RCAS(BP)B without shRNA insert, as a control. Furthermore, uninjected controls of the same stage were kept along with the virus-injected animals. The eggs were further incubated and the embryos were sacrificed by decapitation at E14. BrdU injections were performed according to (Striedter and Keefer, 2000), shortly, BrdU (Sigma) was injected at a concentration of 20 μg/μl into one of the embryos vitelline veins. The embryos were further incubated for 3 hours before fixation at E7 and E9, or until E14 after injection at E7 to cover the entire time span of PCD.

### Fixation and Histology

At the desired stage, embryos were sacrificed by decapitation and the heads were fixed in 4% paraformaldehyde overnight, dehydrated in ascending ethanol concentrations and embedded in paraffin. Ten micrometer microtome sections were collected for hematoxylin and eosin (HE) staining and immunohistochemistry. Neurons were counted on every tenth HE-stained section as described (Oppenheim et al., 1989).

### Immunohistochemistry

Sections were de-paraffinized and heated in citrate buffer for improved antigen retrieval and further incubated with anti-islet-1/2 (40.2D6) 1:50; anti-gag (AMV3C2, 1:200; both from Developmental Studies Hybridoma Bank, University of Iowa), anti-BrdU (Sigma; 1:1000); anti-active-caspase-3 (R&D; 1:500) and visualized using biotinylated secondary antibodies (donkey-anti-mouse; -anti-rabbit; -anti-rat; 1:100; Dianova, Hamburg, Germany) and diaminobenzidine (Vectastain Peroxidase ABC-kit 6100, Vector Laboratories, CA, United States).

### *In Situ* Hybridization

Sections were de-paraffinized and *in situ* hybridization and preparation of digoxigenin-labeled probes for chicken BID and BCL-XL were performed as described previously (Ernsberger et al., 1997). Chicken BID was cloned by RT-PCR using the pGEM-T vector system (Promega, Mannheim, Germany) following the manufacturer’s instruction. The sequences of the primers were: BID forward: TCTGCTAAGATACCTTTTCTGCTCA; BID reverse: CTGCTACCAAAAAGGAGAGGGA. The probe against chicken BCL-XL was made using the chicken EST clone ChEST202O3 from the EST library of the University of Manchester, United Kingdom (Boardman et al., 2002).

## Results

### Analysis of Transcriptome Dynamics of Ciliary Ganglion Development

**FIGURE 1:**
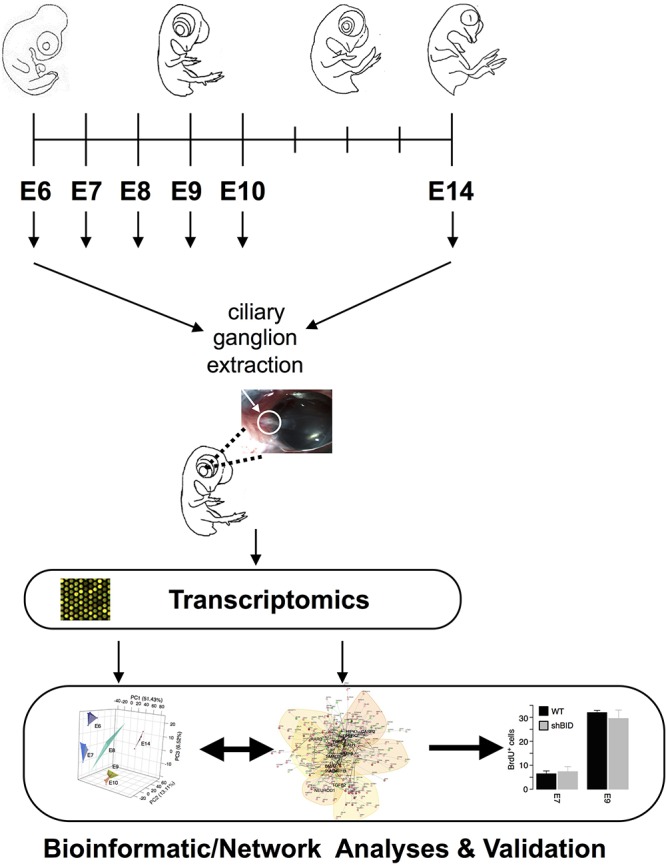
Experimental and analysis Workflow. Ciliary ganglia were dissected from E6–E10 and E14 chicken embryos. Transcriptome analyses were performed from all different time points and were inferred into an apoptotic driven network.

**FIGURE 2:**
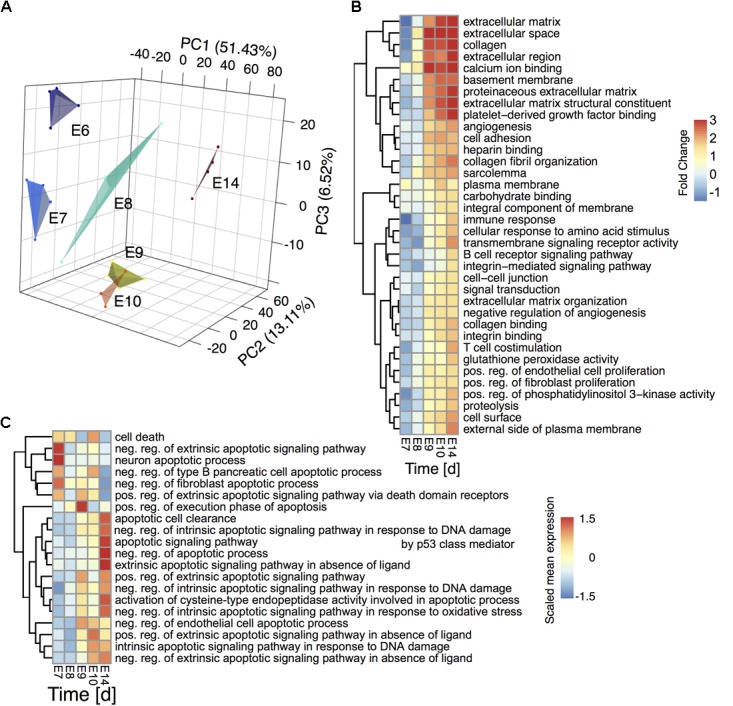
Transcriptome timeseries reveal sequential pathway activity and transient apoptosis during CG development **(A)** Principal component analysis of the ciliary ganglion neuron transcriptomes during development. The plot depicts the first three principal components accounting for ∼51%, ∼13%, and ∼7% of the variance, respectively. A convex hull encloses the individual samples per time point to guide the eye. **(B)** GSEA of the developmental gene expression time series. The heatmap depicts the fold change of the mean statistics of all chicken Gene ontology (GO) terms relative to E6 that are differentially regulated over time (FDR-corrected *q*-value < 0.001). **(C)** GSEA of significantly altered apoptosis and cell death related GO terms over time (*p*-value < 0.1) The heatmap depicts the scaled mean expression per gene set. Rows are hierarchically clustered by their Euclidean distance using the complete linkage method.

To examine the progression of CG neuron development we performed a time series analysis of the transcriptomes from days E6 to E14, taking samples from four biological independent replicates at E6, E7, E8, E9, E10, and E14 using Agilent Gene Expression 4x44K microarrays for network analysis as shown in **Figure 1**. A PCA revealed the dynamic changes of developmental progression of CG (**Figure 2A**). Developmental stages clearly separated along the first principal component (PC1). Moreover, samples at E8/E9 and E9/E10 were displaced along PC2 and PC3, respectively, indicating transient gene regulation processes on these days. The PCA furthermore showed one of the E10 samples as outlier (Supplementary Figure 1, E10 number 1) for unknown biological or technical reasons. We therefore excluded this sample from further analysis. Time-resolved GSEA (Luo et al., 2009) using the chicken GO as gene sets showed a sudden up-regulation of various sets starting at E8 and E9 (**Figure 2B**). Pathways included extracellular processes like cell adhesion, integrin signaling, collagen organization as well as angiogenesis that show their major increase at the end of the developmental stage. This was in line with a previous study on synapse formation in the chicken CG between E5 and E17 (Brusés, 2010), which found a massive change in gene regulation during the maturation phase between E10 and E17. Apparently, a specific temporal gene regulatory sequence followed by pathway activation is a prerequisite for CG development. In this context, several pathways are known to be crucial during neurogenesis, such as Notch-Delta signaling (Cau and Blader, 2009), controlling the expression of proneural genes and the competence to respond to the Jak-Stat (Cau and Blader, 2009), Wnt- (Ciani and Salinas, 2005) and the Sonic Hedgehog pathway (Matise and Wang, 2011). Cell-fate specification is mediated by growth factor signaling, including TGF-beta family members, neurotrophins and neurokines (Bibel and Barde, 2000; Böttner et al., 2000; Krieglstein et al., 2011). This time dependence was also reflected in the change of the mean expression of TGF-beta, Notch and Jak-Stat related gene sets in our experimental setup (Supplementary Figure 2A). All were activated between E9 and E14. On the other hand, neurogenesis related gene function as well as down-regulated gene sets showed a continuous, instead of a sudden de-regulation between E6 and E14. Loss of “stem cell maintenance,” “sympathetic nervous system development” or gain in “Axonogenesis” indicated a steady differentiation of CG neurons, superseding the transient or sudden changes in gene expression described above (Supplementary Figures 2A,B, labeled in red).

### Regulation of Ontogenetic Cell Death

**FIGURE 3:**
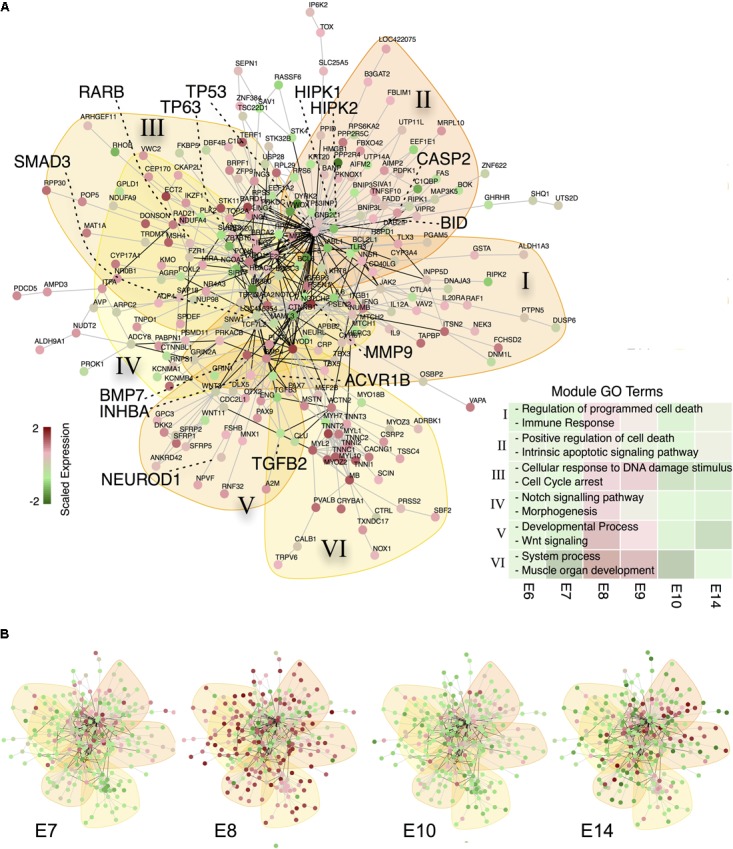
A subgraph of the chicken protein interaction reveals TP53 as central gene of apoptosis during CG development **(A)** Protein-interaction subgraph constructed from genes that are (i) transiently expressed and (ii) apoptosis related. Edges correspond to protein interactions, while node colors depict the expression of each gene scaled along the time points. Colored backdrops indicate significant network modules (*p* < 0.001) calculated from a spin-glass model and simulated annealing. Genes discussed in the text are highlighted by big fonts. The bottom right list depicts representative GO terms enriched in the modules (marked by roman numerals) and the mean scaled gene expression per module as backdrop. **(B)** Relative gene expression over time for the apoptosis network from **(A)**.

As Notch and TGF-beta pathways are closely linked to regulated cell death during CG development we elucidated the apoptosis dynamics between E6 and E14. A hierarchical clustering of all significantly regulated apoptosis or cell death related GO gene sets revealed a transition from the initiation of neuronal apoptotic processes on E7 via a cell death execution phase on E8 to apoptotic cell clearance at E14 (**Figure 2C**). The analysis further showed a shift between intrinsic and extrinsic regulation of apoptotic events from E7 to E9. To elucidate key molecular players of this transient apoptosis process, we constructed a maximum scoring subgraph (Fang and Gough, 2014) from the STRING chicken interactome (Franceschini et al., 2013). With the goal not only to find the apoptosis related genes, but also to find the pathways through which they are connected we identified transiently expressed genes related to apoptosis by first fitting a second order polynomial to each gene time series and assigning the quadratic coefficient as putative subgraph score. To this score we added a positive or respectively, negative weight, if the gene was or was not related to apoptosis signaling. Next, a heuristic search algorithm deduced a fully connected subgraph from the chicken interactome that included a maximal number of genes having a positive and a minimal number of genes having negative node scores. The number of nodes in the induced subgraph scaled inversely with the size of the positive/negative weights added to the subgraph scores (Supplementary Figure 3A). As a compromise between specificity and size of the subgraph we chose a score penalty of two, which resulted in a highly-modularized CG apoptosis network consisting of 259 nodes and 602 edges (**Figure 3A**, and Supplementary Table 1) having long-tailed node degree distribution (Supplementary Figure 3B). A hypergeometric test showed a functional enrichment of apoptosis (Modules I and II), DNA damage (Module III), Notch and Wnt-signaling (Modules IV-V) as well as development (Modules IV-VI). Both network modules and gene expression showed a clear maximum at E8/E9 (GO terms backdrop **Figures 2**, **3B**) and furthermore at E14, stemming from apoptotic cell clearance.

**FIGURE 4:**
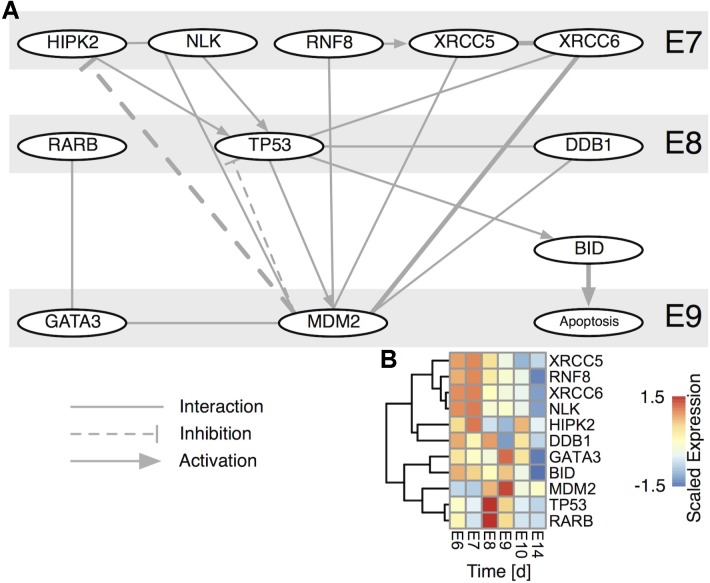
TP53 centered subnetwork derived from the STRING interaction database. **(A)** The network comprises all nodes having a direct interaction with TP53 and MDM2 for either chicken (thick edges) or human. Edges are directed, if there is a known such interaction. Nodes are time-ordered from top to bottom. **(B)** Scaled expression of genes in **(A)**. Rows are hierarchically clustered by their Euclidean distance using the complete linkage method.

Based on this informative CG apoptosis network we were able to calculate the network betweenness and eigenvector centrality that determine which nodes are central or peripheral to the network (Supplementary Figure 3C). The most central nodes were the transcription factors TP53 and TP63 both of which were transiently up-regulated at E8 (Supplementary Figure 4A). Also central to the network were members of the TGF-beta superfamily signaling pathways, such as TGFB2 (Transforming Growth Factor, Beta 2), SMAD3 (SMAD Family Member 3) or ACVR1B (Activin A Receptor, Type IB) confirming the importance of activin signaling in CG development (Supplementary Figure 4B) (Darland and Nishi, 1998). Among the most central, yet transiently expressed genes at E8/E9, we find MMP9 (Matrix Metallopeptidase 9) and BMP7 (Bone Morphogenetic Protein 7), confirming their essential role in neuronal cell death (Murase and McKay, 2012) and early embryogenesis (Trousse et al., 2001). Genes at the network periphery possess a low centrality and possibly serve as upstream activators or downstream effectors. In fact, such nodes are the TP53 cofactors HIPK1 and 2 (Homeodomain Interacting Protein Kinase 1 and 2) that already transiently peak at E7 (Akaike et al., 2014), RARB, whose involvement in CG development we recently showed (Koszinowski et al., 2015), NEUROD1 (Neuronal Differentiation 1), inducing terminal differentiation (Boutin et al., 2010) and lastly BID, which is known to induce apoptosis through activation of TP53 (Haupt et al., 2003). This prompted us to construct a subnetwork around TP53 (**Figure 4A**). BID is induced by TP53 and indeed showed its peak regulation at E9 (**Figure 4B**). Moreover, several TP53 stabilizing/activation factors were expressed at E7 already (**Figure 4B**), namely the pro-apoptotic activators HIPK2, NLK (Nemo-like Kinase) and RNF8 (Ring Finger Protein 8). NLK is required for p53 activation, as it stabilizes p53 by blocking MDM2 (transformed mouse 3T3 cell double minute 2) mediated degradation (Zhang et al., 2014). RARB indirectly interacts with TP53 via GATA3 (GATA binding protein 3) and MDM2, thereby modulating the time-dependent apoptotic effect. Thus, the network corroborated a tight transcriptional regulation of TP53, already initiated at E7 that predicted a defined temporal window of BID-mediated apoptosis.

### Suppression of BID During Ciliary Ganglion Development Prevents Ontogenetic Cell Death

**FIGURE 5:**
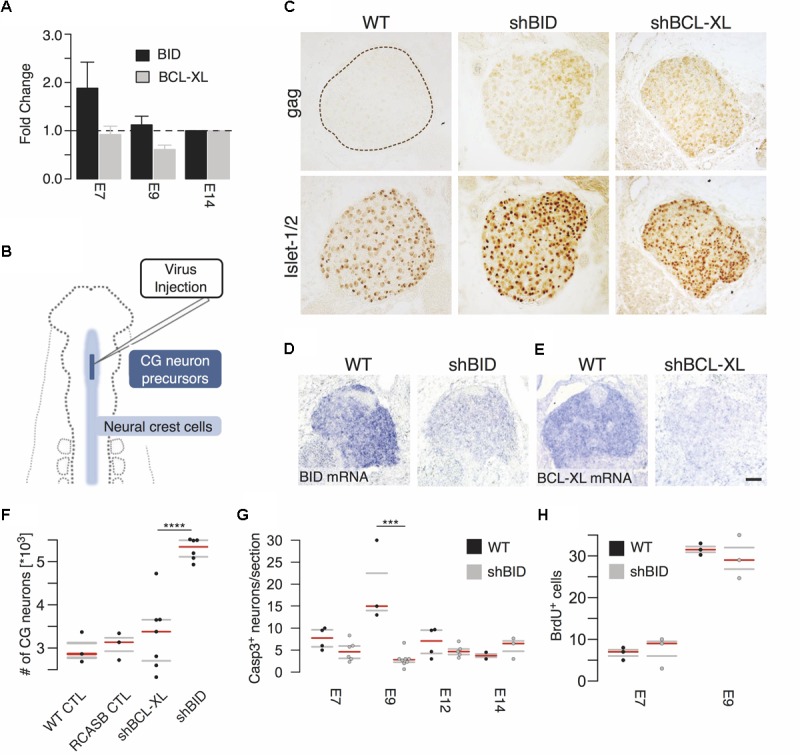
Virus-mediated knockdown of BID and BCL-XL during ciliary ganglion development. **(A)** Time course of BID and BCL-XL expression during ciliary ganglion development normalized to E14 assessed by RT-qPCR. **(B)** Diagram of virus-mediated RNAi of BID and BCL-XL. Virus concentrate is injected into the neural tube of HH9 chicken embryos to infect the ciliary ganglion neuron precursors. **(C)** Representative pictures of the uninfected wild type control (WT) and virus-infected ganglia (gag). Islet1/2 staining identifies all neurons of the ganglion. shBID and shBCL-XL depict the respective virus knockdown constructs. **(D,E)** *In situ* hybridization for BID and BCL-XL mRNA after knockdown relative to WT (scale bar = 50 μm). **(F)** Increase in the number of CG neurons at E14 after knockdown of BID. (*n*(CTL) = 3; *n*(RCASB CTL) = 3; *n*(shBID) = 6; *n*(shBCL-XL = 7; ^∗∗∗∗^*p* < 0.0001, two-sided *t*-test). **(G)** Number of caspase-3 positive neurons after BID knockdown. (*n*(E9 CTL) = 3; *n*(E9 shBID) = 8, ^∗∗∗^*p* = 0.0005, two-sided *t*-test). **(H)** BrdU positive cells after BID knockdown relative to WT (*n*(E9 CTL) = 3; *n*(E9 shBID) = 3). Horizontal bars denote the 0.25/0.75 and 0.5 quantiles (median) of the biologically independent replicates in gray and red, respectively.

Having demonstrated the close dependence of BID on TP53 we next tested *in vivo* by RNAi knockdown its significance in the regulation of apoptotic events during CG development. As control, we chose a second member of the Bcl-2 protein family BCL-XL (BCL2L1), which has anti-apoptotic function (Boise et al., 1993). First, we confirmed the expression of BID and BCL-XL via RT-qPCR (**Figure 5A**). BCL-XL showed a local expression minimum around E9, while BID expression decreased from E7 compared to E14. This is in line with the expression timeseries from the microarray data, which showed additionally a transient maximum around E9 (**Figure 4B**).

For RNAi-mediated knockdown, RCAS(BP)B-plasmids carrying the micro-RNA operon expression cassettes with the shRNA-hairpin insert against BID (RCASBshBID) and BCL-XL (RCASBshBCL-XL) were generated (Das et al., 2006). The virus-concentrate was injected into the neural tube of HH9 (E2.5) chicken embryos at the level of the mesencephalon (**Figure 5B**) to infect CG neuron precursors before their delamination from the neural tube. Approximately 50% of the embryos showed a lasting ganglion infection detectable until E14 (**Figure 5C**, bottom row). Islet1/2 staining identifies all neurons of the ganglion. *In situ* hybridization against BID and BCL-XL mRNA on E7 confirmed the reduction of both BID and BCL-XL mRNA levels (**Figures 5D,E**). As a result of BID knockdown neuron numbers increased by a striking 78% at E14 (5284 ± 101.9, BID knockdown vs. 2973 ± 204.8 wild type control; **Figure 5F**), while the knockdown of BCL-XL did not affect the number of neurons surviving PCD. This increase after BID knockdown was most likely the result of suppressed apoptosis. Active caspase-3 staining revealed a strong and transient peak at E9 in wild type CGs that fits nicely with the dynamic expression network of TP53. In contrast, BID knockdown completely abrogated the peak and hardly any apoptotic neurons were observed at any time point (**Figure 5G**). To exclude increased proliferation as cause for higher neuronal precursors, embryos were injected with BrdU at either E7 or E9 and incubated for an additional 3 h. No impact of BID knockdown on BrdU staining was detectable (**Figure 5H**). Therefore, we concluded that knockdown of BID expression in the developing CG led to an overall deficiency of the neurons to undergo developmental cell death.

## Discussion

The present study elucidated and described molecular networks regulating important aspects of parasympathetic development, using the chicken CG, a well-described classical model system (Dryer, 1994). The genome-wide gene expression analysis covered the entire development of the ganglion between E6 and E14. The precursors of CG neurons migrate from the cranial neural crest towards the eye and coalesce to form the ganglion by E4, whereafter they become postmitotic and set out to express neuronal marker proteins (Landmesser and Pilar, 1972; Rohrer and Thoenen, 1987). Between E6 and E10 efferent and afferent synaptic connections become established, accompanied by a massive wave of neuronal PCD (Landmesser and Pilar, 1974; Narayanan and Narayanan, 1981) and these processes are finished by E14 (Landmesser and Pilar, 1978). CG development undergoes strict and well-defined stages. Functional analysis of the gene expression correlated well with the differentiation progress, however the PCD signature remained weak, probably because only a subpopulation of neurons executes cell death. Still, its importance showed in the up-regulation of several pathways such as TGF-beta, Notch and Jak-Stat at E9 after the transient wave of apoptosis. Due to the temporal transcriptional resolution during CG development, we were only able to perform a network reconstruction and analysis that revealed the concerted regulation of many players of apoptosis, interacting in several modules with TP53 and TP63 as central hub nodes. Indeed, the expression of TP53 was already “prepared” at E7 through activating and stabilizing factors and through the interplay with MDM2. However, we further predicted and verified an important role of the peripheral network nodes, such as RARB and BID, acting as effectors and interfacing the network to the cellular phenotype. Here, the effect of BID silencing *in vivo* corroborates its essential role in neuronal development in a defined time window. BID is known to play a crucial role in the regulation of the mitochondrial cell death checkpoint in neurons and has been suggested as a target for pharmacological therapy (Culmsee and Plesnila, 2006). Moreover, a RIPK1 (Receptor-Interacting Protein Kinase 1) dependent BID cleavage was shown to induce a TNF alpha-dependent cell death (Chen et al., 2015) or the FADD (Fas Associated Via Death Domain)/caspase-8/BID/cytochrome c axis that links death receptors and mitochondria regarding ROS generation and apoptosis (Kim et al., 2017). Both, RIPK1 and FADD, are also integrated in our protein-interaction subgraph like BID within module II that are linked to the GO terms “positive regulation of cell death and intrinsic apoptotic signaling pathway.” Nevertheless, BID-deficient mice do not show neurodevelopmental abnormalities suggesting BID activity compensation by yet unidentified BH3-only proteins, as functional redundancies have been observed among Bcl-2 proteins (Lindsten et al., 2005). Chicken possibly lack a BH3-only protein compensating, since we also couldn’t detect it on transcriptome level in our data set, and may thus fully depend on the presence of BID. In this context, p53-dependent apoptosis mediated by NOXA has been described as the predominant axotomy-induced motor neuron death in mice (Kiryu-Seo et al., 2005). PUMA, but not BIM, has been suggested to induce cell death following p53 activation by 6-OHDA in a cell culture model of Parkinson’s disease (Biswas et al., 2005). PUMA has also been suggested in response to p53 activation to cooperate with BIM in β-amyloid-induced neuronal cell death (Akhter et al., 2014). Furthermore, PUMA or Fas/CD95 knockdown confers neuroprotection in retinal ganglion cell death in rodents (Wilson et al., 2013). However, chicken may not have PUMA or NOXA. On the other hand, PCD in chicken CG development does not seem to rely on the anti-apoptotic function of BCL-XL, as shown in **Figure 5**, suggesting a functional redundancy in these proteins. However, a high level of redundancy among BCL-2 proteins is reducing the impact of BCL-XL knockdown, as previously characterized in mammalian systems. Nevertheless, this analysis is currently not feasible in chicken neurons and pro-survival BCL-2 activities would only be important, if BID is cleaved. We have not been able to demonstrate tBID-induced mitochondrial apoptosis. Therefore, a role of BID in chick neuronal development independent of the BCL-2 protein interplay at the OMM (outer mitochondrial membrane) cannot be excluded and need further evaluations. In summary, we showed how transcriptome time series analysis with network inference can be used to provide new insights into the roles of Bcl-2 family proteins for apoptosis during CG development that was furthermore experimental validated. The altered functional redundancy of these proteins in comparison to mouse corroborated the particular implementation of apoptosis in an avian species highlights the need for further detailed molecular investigation in this key event during neuronal CG development.

## Data Availability Statement

The datasets generated and analyzed for this study can be found in the Gene Expression Omnibus repository under the accession ID GSE65426.

## Ethics Statement

This study was exempted from ethical approval, because we used chicken embryos which are not defined as animals during the used stages E6 to E14.

## Author Contributions

SK and KK planned the experiment. SK and VLP performed the experiments. KK and FE discussed the results and supported article writing. HB and MB analyzed the transcriptome data, composed the network model, made the figures and tables and wrote the article together with SK.

## Conflict of Interest Statement

The authors declare that the research was conducted in the absence of any commercial or financial relationships that could be construed as a potential conflict of interest.

## References

[B1] Akaike Y. Kuwano Y. Nishida K. Kurokawa K. Kajita K. Kano S. (2014). Homeodomain-interacting protein kinase 2 regulates DNA damage response through interacting with heterochromatin protein 1γ. *Oncogene* 34 3463–3473. 10.1038/onc.2014.278 25151962

[B2] Akhter R. Sanphui P. Biswas S. C. (2014). The essential role of p53-up-regulated modulator of apoptosis (Puma) and its regulation by FoxO3a transcription factor in β-amyloid-induced neuron death. *J. Biol. Chem.* 289 10812–10822. 10.1074/jbc.M113.519355 24567336 PMC4036450

[B3] Bibel M. Barde Y. A. (2000). Neurotrophins: key regulators of cell fate and cell shape in the vertebrate nervous system. *Genes Dev.* 14 2919–2937. 10.1101/gad.841400 11114882

[B4] Biswas S. C. Ryu E. Park C. Malagelada C. Greene L. A. (2005). Puma and p53 play required roles in death evoked in a cellular model of Parkinson disease. *Neurochem. Res.* 30 839–845. 10.1007/s11064-005-6877-5 16187218

[B5] Boardman P. E. Sanz-Ezquerro J. Overton I. M. Burt D. W. Bosch E. Fong W. T. (2002). A comprehensive collection of chicken cDNAs. *Curr. Biol.* 12 1965–1969. 10.1016/S0960-9822(02)01296-4 12445392

[B6] Boise L. H. González-García M. Postema C. E. Ding L. Lindsten T. Turka L. A. (1993). bcl-x, a bcl-2-related gene that functions as a dominant regulator of apoptotic cell death. *Cell* 74 597–608. 10.1016/0092-8674(93)90508-N 8358789

[B7] Böttner M. Krieglstein K. Unsicker K. (2000). The transforming growth factor-betas: structure, signaling, and roles in nervous system development and functions. *J. Neurochem.* 75 2227–2240. 10.1046/j.1471-4159.2000.0752227.x 11080174

[B8] Boutin C. Hardt O. de Chevigny A. Coré N. Goebbels S. Seidenfaden R. (2010). NeuroD1 induces terminal neuronal differentiation in olfactory neurogenesis. *Proc. Natl. Acad. Sci. U.S.A.* 107 1201–1206. 10.1073/pnas.0909015107 20080708 PMC2824315

[B9] Brusés J. L. (2010). Identification of gene transcripts expressed by postsynaptic neurons during synapse formation encoding cell surface proteins with presumptive synaptogenic activity. *Synapse* 64 47–60. 10.1002/syn.20702 19728367 PMC2783745

[B10] Cau E. Blader P. (2009). Notch activity in the nervous system: to switch or not switch? *Neural Develop.* 4 36. 10.1186/1749-8104-4-36 19799767 PMC2761386

[B11] Chen C. Grennan K. Badner J. Zhang D. Gershon E. Jin L. (2011). Removing batch effects in analysis of expression microarray data: an evaluation of six batch adjustment methods. *PLoS One* 6:e17238. 10.1371/journal.pone.0017238 21386892 PMC3046121

[B12] Chen G. Cheng X. Zhao M. Lin S. Lu J. Kang J. (2015). RIP1-dependent Bid cleavage mediates TNFα-induced but Caspase-3-independent cell death in L929 fibroblastoma cells. *Apoptosis* 20 92–109. 10.1007/s10495-014-1058-0 25398540

[B13] Ciani L. Salinas P. C. (2005). WNTS in the vertebrate nervous system: from patterning to neuronal connectivity. *Nat. Rev. Neurosci.* 6 351–362. 10.1038/nrn1665 15832199

[B14] Csardi G. Nepusz T. (2006). The Igraph Software Package for Complex Network Research. Available at: http://igraph.org

[B15] Culmsee C. Plesnila N. (2006). Targeting Bid to prevent programmed cell death in neurons. *Biochem. Soc. Trans.* 34 1334–1340. 10.1042/BST0341334 17073814

[B16] Darland D. C. Nishi R. (1998). Activin A and follistatin influence expression of somatostatin in the ciliary ganglion in vivo. *Dev. Biol.* 202 293–303. 10.1006/dbio.1998.8998 9769180

[B17] Das R. M. Van Hateren N. J. Howell G. R. Farrell E. R. Bangs F. K. Porteous V. C. (2006). A robust system for RNA interference in the chicken using a modified microRNA operon. *Dev. Biol.* 294 554–563. 10.1016/j.ydbio.2006.02.020 16574096

[B18] Dryer S. E. (1994). Functional development of the parasympathetic neurons of the avian ciliary ganglion: a classic model system for the study of neuronal differentiation and development. *Prog. Neurobiol.* 43 281–322. 10.1016/0301-0082(94)90003-5 7816929

[B19] Egawa R. Hososhima S. Hou X. Katow H. Ishizuka T. Nakamura H. (2013). Optogenetic probing and manipulation of the Calyx-type presynaptic terminal in the embryonic chick ciliary ganglion. *PLoS One* 8:e59179. 10.1371/journal.pone.0059179 23555628 PMC3605445

[B20] Ernsberger U. Patzke H. Rohrer H. (1997). The developmental expression of choline acetyltransferase (ChAT) and the neuropeptide VIP in chick sympathetic neurons: evidence for different regulatory events in cholinergic differentiation. *Mech. Dev.* 68 115–126. 10.1016/S0925-4773(97)00135-4 9431809

[B21] Fang H. Gough J. (2014). The ‘dnet’ approach promotes emerging research on cancer patient survival. *Genome Med.* 6:64. 10.1186/s13073-014-0064-8 25246945 PMC4160547

[B22] Franceschini A. Szklarczyk D. Frankild S. Kuhn M. Simonovic M. Roth A. (2013). STRING v9.1: protein-protein interaction networks, with increased coverage and integration. *Nucleic Acids Res.* 41 D808–D815. 10.1093/nar/gks1094 23203871 PMC3531103

[B23] Hamburger V. Hamilton H. L. (1992). A series of normal stages in the development of the chick embryo. 1951. *Dev. Dyn.* 195 231–272. 10.1002/aja.1001950404 1304821

[B24] Haupt S. Berger M. Goldberg Z. Haupt Y. (2003). Apoptosis - the p53 network. *J. Cell Sci.* 116 4077–4085. 10.1242/jcs.00739 12972501

[B25] Kim W. S. Lee K. S. Kim J. H. Kim C. K. Lee G. Choe J. (2017). The caspase-8/Bid/cytochrome c axis links signals from death receptors to mitochondrial reactive oxygen species production. *Free Radic. Biol. Med.* 112 567–577. 10.1016/j.freeradbiomed- 28888620

[B26] Kiryu-Seo S. Hirayama T. Kato R. Kiyama H. (2005). Noxa is a critical mediator of p53-dependent motor neuron death after nerve injury in adult mouse. *J. Neurosci.* 25 1442–1447. 10.1523/JNEUROSCI.4041-04.2005 15703398 PMC6726006

[B27] Koszinowski S. Boerries M. Busch H. Krieglstein K. (2015). RARβ regulates neuronal cell death and differentiation in the avian ciliary ganglion. *Dev. Neurobiol.* 75 1204–1218. 10.1002/dneu.22278 25663354 PMC4832352

[B28] Krieglstein K. Zheng F. Unsicker K. Alzheimer C. (2011). More than being protective: functional roles for TGF-β/activin signaling pathways at central synapses. *Trends Neurosci.* 34 421–429. 10.1016/j.tins.2011.06.002 21742388

[B29] Landmesser L. Pilar G. (1972). The onset and development of transmission in the chick ciliary ganglion. *J. Physiol.* 222 691–713. 10.1113/jphysiol.1972.sp009822 4338175 PMC1331408

[B30] Landmesser L. Pilar G. (1974). Synaptic transmission and cell death during normal ganglionic development. *J. Physiol.* 241 737–749. 10.1113/jphysiol.1974.sp010681 4373568 PMC1331060

[B31] Landmesser L. Pilar G. (1978). Interactions between neurons and their targets during in vivo synaptogenesis. *Fed. Proc.* 37 2016–2022. 205438

[B32] Lindsten T. Zong W.-X. Thompson C. B. (2005). Defining the role of the Bcl-2 family of proteins in the nervous system. *Neuroscientist* 11 10–15. 10.1177/1073858404269267 15632274

[B33] Luo W. Friedman M. S. Shedden K. Hankenson K. D. Woolf P. J. (2009). GAGE: generally applicable gene set enrichment for pathway analysis. *BMC Bioinformatics* 10:161. 10.1186/1471-2105-10-161 19473525 PMC2696452

[B34] Matise M. P. Wang H. (2011). Sonic hedgehog signaling in the developing CNS where it has been and where it is going. *Curr. Top. Dev. Biol.* 97 75–117. 10.1016/B978-0-12-385975-4.00010-3 22074603

[B35] Murase S. McKay R. D. (2012). Matrix metalloproteinase-9 regulates survival of neurons in newborn hippocampus. *J. Biol. Chem.* 287 12184–12194. 10.1074/jbc.M111.297671 22351756 PMC3320970

[B36] Narayanan Y. Narayanan C. H. (1981). Ultrastructural and histochemical observations in the developing iris musculature in the chick. *J. Embryol. Exp. Morphol.* 62 117–127. 7276805

[B37] Oppenheim R. W. Cole T. Prevette D. (1989). Early regional variations in motoneuron numbers arise by differential proliferation in the chick embryo spinal cord. *Dev. Biol.* 133 468–474. 10.1016/0012-1606(89)90050-X 2731638

[B38] Rohrer H. Thoenen H. (1987). Relationship between differentiation and terminal mitosis: chick sensory and ciliary neurons differentiate after terminal mitosis of precursor cells, whereas sympathetic neurons continue to divide after differentiation. *J. Neurosci.* 7 3739–3748. 10.1523/JNEUROSCI.07-11-03739.1987 3681410 PMC6569030

[B39] Ruffinatti F. A. Gilardino A. Lovisolo D. Ferraro M. (2013). Spatial wavelet analysis of calcium oscillations in developing neurons. *PLoS One* 8:e75986. 10.1371/journal.pone.0075986 24155880 PMC3796547

[B40] Simpson J. Keefe J. Nishi R. (2013). Differential effects of RET and TRKB on axonal branching and survival of parasympathetic neurons. *Dev. Neurobiol.* 73 45–59. 10.1002/dneu.22036 22648743 PMC4037150

[B41] Smyth G. K. (2005). “Limma: linear models for microarray data,” in *Bioinformatics and Computational Biology Solutions Using R and Bioconductor*, eds Gentleman R. Carey V. Dudoit S. Irizarry R. Huber W. (New York, NY: Springer), 397–420. 10.1007/0-387-29362-0_23

[B42] Stanchev D. Sargent P. B. (2011). α7-Containing and non-α7-containing nicotinic receptors respond differently to spillover of acetylcholine. *J. Neurosci.* 31 14920–14930. 10.1523/JNEUROSCI.3400-11.2011 22016525 PMC3342687

[B43] Striedter G. F. Keefer B. P. (2000). Cell migration and aggregation in the developing telencephalon: pulse-labeling chick embryos with bromodeoxyuridine. *J. Neurosci.* 20 8021–8030. 10.1523/JNEUROSCI.20-21-08021.2000 11050123 PMC6772730

[B44] Trousse F. Esteve P. Bovolenta P. (2001). Bmp4 mediates apoptotic cell death in the developing chick eye. *J. Neurosci.* 21 1292–1301. 10.1523/JNEUROSCI.21-04-01292.2001 11160400 PMC6762245

[B45] Wang K. Yin X. M. Chao D. T. Milliman C. L. Korsmeyer S. J. (1996). BID: a novel BH3 domain-only death agonist. *Genes Dev.* 10 2859–2869. 10.1101/gad.10.22.2859 8918887

[B46] Wilson A. M. Morquette B. Abdouh M. Unsain N. Barker P. A. Feinstein E. (2013). ASPP1/2 regulate p53-dependent death of retinal ganglion cells through PUMA and Fas/CD95 activation in vivo. *J Neurosci.* 33 2205–2216. 10.1523/JNEUROSCI.2635-12 23365256 PMC6619125

[B47] Zamburlin P. Ruffinatti F. A. Gilardino A. Farcito S. Parrini M. Lovisolo D. (2013). Calcium signals and FGF-2 induced neurite growth in cultured parasympathetic neurons: spatial localization and mechanisms of activation. *Pflügers Arch.* 465 1355–1370. 10.1007/s00424-013-1257-5 23529843

[B48] Zhang H.-H. Li S.-Z. Zhang Z.-Y. Hu X.-M. Hou P.-N. Gao L. (2014). Nemo-like kinase is critical for p53 stabilization and function in response to DNA damage. *Cell Death Differ.* 21 1656–1663. 10.1038/cdd.2014.78 24926618 PMC4158690

