# Supplementary material for: Bid Expression Network Controls Neuronal Cell Fate During Avian Ciliary Ganglion Development
Source: Front Physiol. 2018 Jun 29;9:797. doi: 10.3389/fphys.2018.00797 (PMC6034111; doi:10.3389/fphys.2018.00797)
Supplement: Supplementary file 2 [file Data_Sheet_1.PDF]

Supplementary Material

Bid Expression Network Controls Neuronal Cell Fate During Avian Ciliary Ganglion Development

Sophie Koszinowski, Veronica La Padula, Frank Edlich, Kerstin Krieglstein, Hauke Busch[4], Melanie Boerries[*]

*** Correspondence:**
m.boerries@dkfz.de

1 Supplementary Figures and Tables

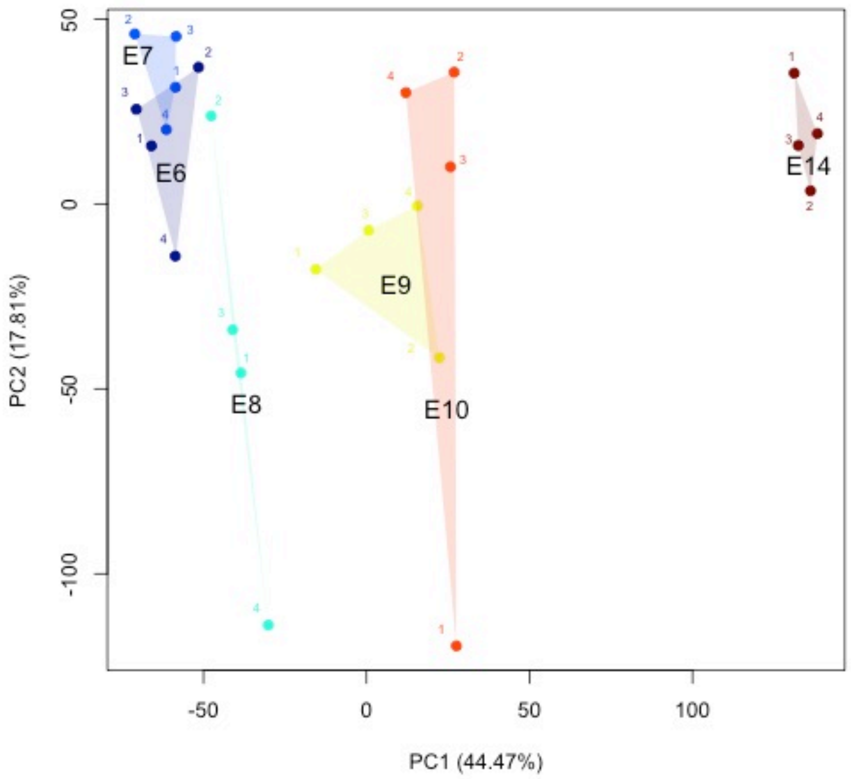

Supplementary Figure 1. Principal component analysis of the ciliary ganglion neuron transcriptomes during development. The plot depicts the first two principal components using all samples accounting for ~44%, ~18% of the variance, respectively. A convex hull encloses the individual samples per time point to guide the eye. Small numbers indicate the sample replicates. Replicate 1 among the E10 samples has been discarded from further analysis as a clear outlier.

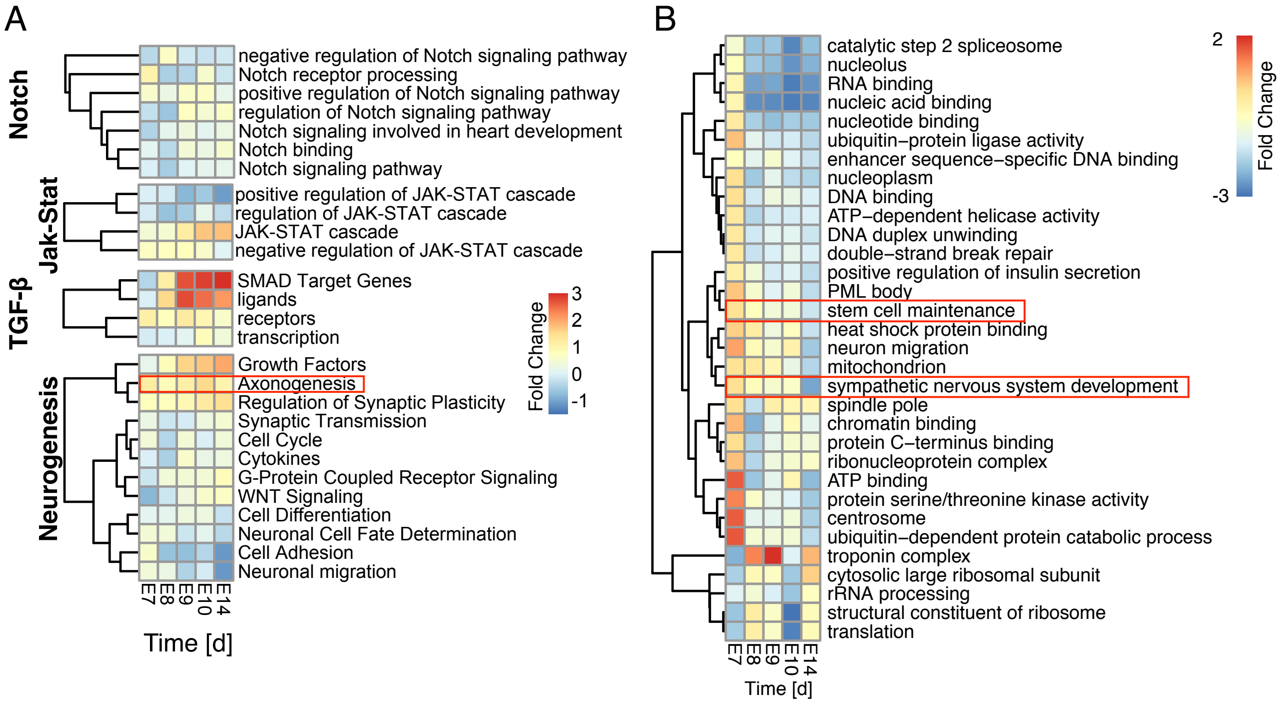

Supplementary Figure 2. Gene Set Enrichment Analysis (GSEA) of the developmental gene expression time series. (A) Heatmap depicting Neurogenesis, TGF-beta and Jak-Stat and Notch signaling related genes. Gene sets for analysis were obtained from RT2 RNA QC PCR Array® (SaBiosciences, Qiagen) gene panels and translated to their chicken orthologs using BiomaRt. (B) The heatmap depicts the fold change of all chicken Gene ontology (GO) terms relative to E6 that were differentially regulated over time (FDR-corrected q-value < 0.01 for at least one timepoint) and preferentially down-regulated. The heatmap rows show the scaled means of the test statistics from the GSEA analysis using the R/Bioconductor gage library.

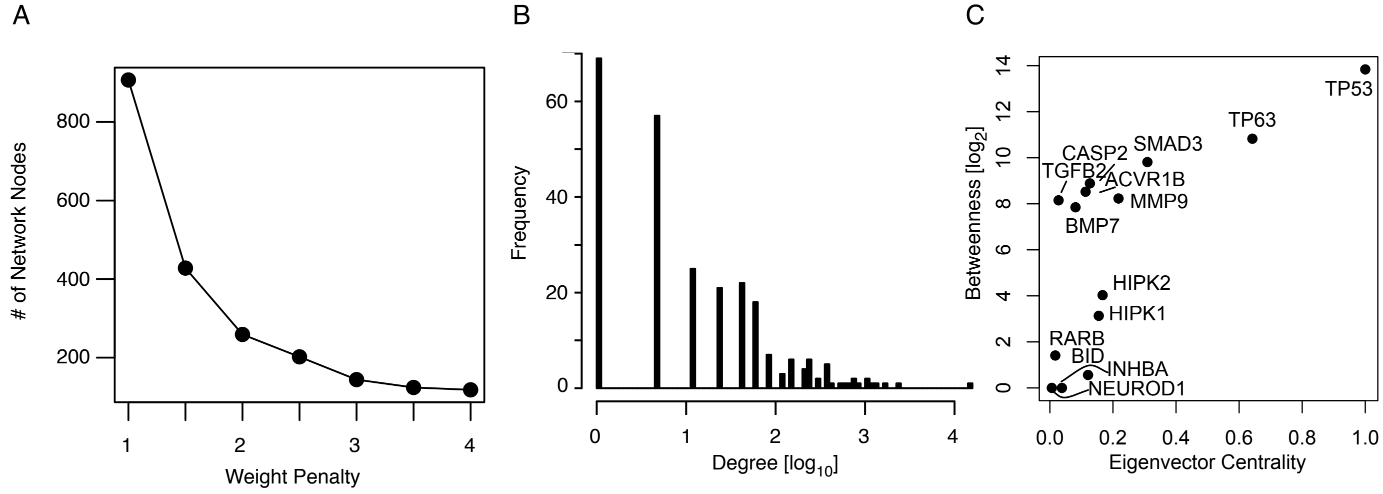

Supplementary Figure 3. (A) Size of the induced apoptosis subnetwork as a function of the weight penalty added to the gene score, i.e. the value added (subtracted) to the gene score, if the gene has (does not have) apoptotic function. The network size increases sharply, if the penalty drops below 2. **(B)** Degree distribution of the 259 network nodes from Fig. 2A. The distribution shows a long-tailed distribution with many nodes having few and few nodes having many edges, acting as network hubs. **(C)** Eigenvector centrality and Betweenness of selected nodes from the apoptosis network in Fig. 3A. Network properites have been calculated using the igraph library in R. The Betweenness is a measure of the number of shortest paths between any two nodes that go through a particular node. High betweenness nodes preferentially connect network modules. The eigenvector centrality denotes the extent by which a node is linked to highly connected parts of the network and is a measure of the influence of a node in a network.

A

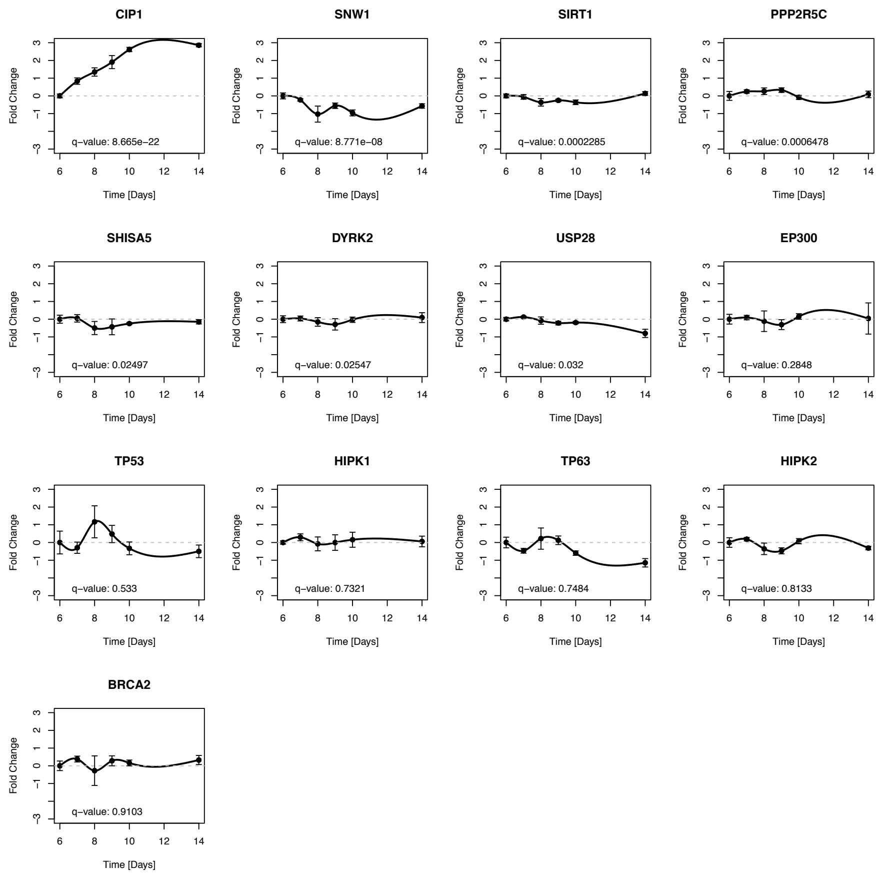
CIP1
q-value: 8.665e-22
SNW1
q-value: 8.771e-08
SIRT1
q-value: 0.0002285
PPP2R5C
q-value: 0.0006478
SHISA5
q-value: 0.02497
DYRK2
q-value: 0.02547
USP28
q-value: 0.032
EP300
q-value: 0.2848
TP53
q-value: 0.533
HIPK1
q-value: 0.7321
TP63
q-value: 0.7484
HIPK2
q-value: 0.8133
BRCA2
q-value: 0.9103
Fold Change
Time [Days]

B

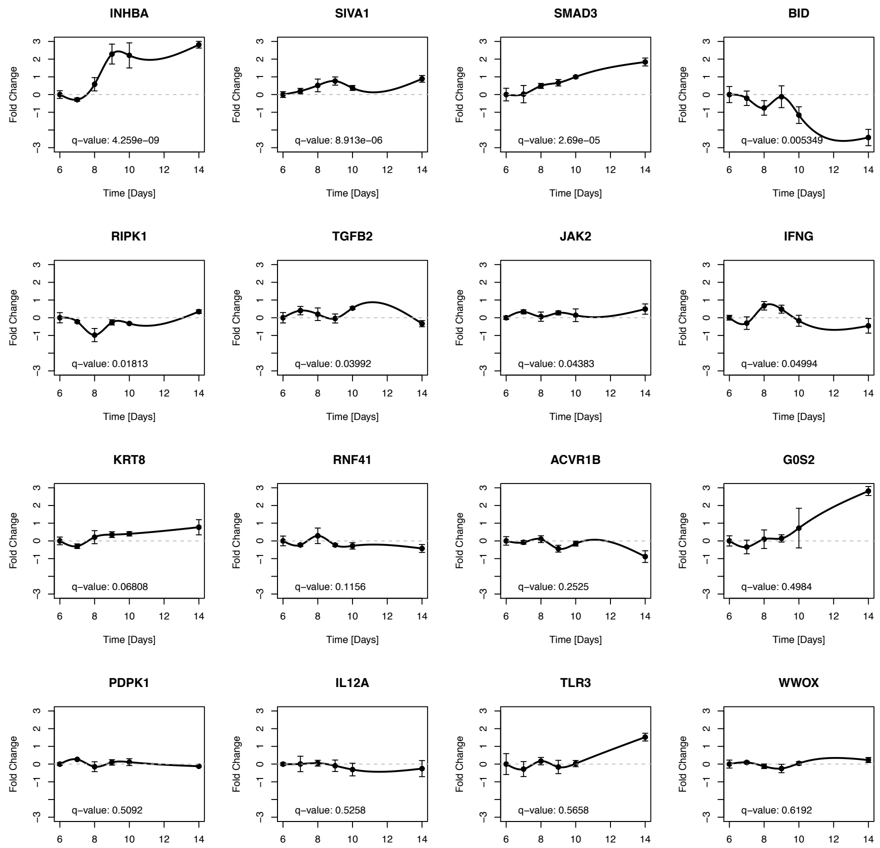
INHBA
q-value: 4.259e-09
SIVA1
q-value: 8.913e-06
SMAD3
q-value: 2.69e-05
BID
q-value: 0.005349
RIPK1
q-value: 0.01813
TGFB2
q-value: 0.03992
JAK2
q-value: 0.04383
IFNG
q-value: 0.04994
KRT8
q-value: 0.06808
RNF41
q-value: 0.1156
ACVR1B
q-value: 0.2525
G0S2
q-value: 0.4984
PDPK1
q-value: 0.5092
IL12A
q-value: 0.5258
TLR3
q-value: 0.5658
WWOX
q-value: 0.6192
Fold Change
Time [Days]

Supplementary Figure 4. (A) Fold change expression over time of genes from the GO-Term "intrinsic apoptotic signaling pathway in response to DNA damage by p53 class mediator" relative to E6. **(B)** Fold change expression over time of genes from the GO-Term "extrinsic apoptotic signaling pathway". Expression values are interpolated by cubic splines to guide the eye. Error bars denote the standard deviation. The inserts depict the q-value of differential regulation of the gene over time obtained from an analysis of deviance [1].

Supplementary Table 1 Conditional Hypergeometric test of GO enrichment of genes present in the significant network modules (Fig. 2B). Conditional Enrichment was calculated using the R library GOstats [2] and tested against all genes present in the STRING-deduced chicken interactome. The work sheets list all significantly enriched biological processes (BP) GO terms for each module (p-value < 0.01). The columns denote, in this order, the GO id, the enrichment p-value, the Odds ratio for each category term, an indicator of the level of gene enrichment within the module against all genes, Expected Count: expected number of genes in the selected network module for each tested category, Counts: number of genes in the selected module that are annotated for the GO category, Size: number of genes among all genes annotated for the GO category. Term: name of the GO term

Supplementary File 1 GraphML file [3] of the apoptosis network in Fig. 3. The file contains all node and edge information together with the gene names, Entrez IDs as well as the node scores for the network reconstruction and edge scores from the STRING database.

References

Brandes, U., Eiglsperger, M., Lerner, M., and Pich, C. (2013). "Graph Markup Language (GraphML)," in *Handbook of Graph Drawing and Visualization* (CRC Press), 517–541.

Busch, H., Boerries, M., Bao, J., Hanke, S. T., Hiss, M., Tiko, T., et al. (2013). Network Theory Inspired Analysis of Time-Resolved Expression Data Reveals Key Players Guiding P. patens Stem Cell Development. *PLoS ONE* 8, e60494. doi:10.1371/journal.pone.0060494.

Falcon, S., and Gentleman, R. (2007). Using GOstats to test gene lists for GO term association. *Bioinforma. Oxf. Engl.* 23, 257–258. doi:10.1093/bioinformatics/btl567.
